# The Temperature Feature of ChatGPT: Modifying Creativity for Clinical Research

**DOI:** 10.2196/53559

**Published:** 2024-03-08

**Authors:** Joshua Davis, Liesbet Van Bulck, Brigitte N Durieux, Charlotta Lindvall

**Affiliations:** 1 Department of Psychosocial Oncology and Palliative Care, Dana-Farber Cancer Institute Boston, MA United States; 2 Albany Medical College Albany, NY United States; 3 KU Leuven Department of Public Health and Primary Care, KU Leuven-University of Leuven Leuven Belgium; 4 Research Foundation Flanders (FWO) Brussels Belgium; 5 Department of Medicine, Brigham and Women's Hospital Boston, MA United States; 6 Harvard Medical School, Harvard University Boston, MA United States

**Keywords:** artificial intelligence, ChatGPT, clinical communication, creative, creativity, customization, customize, customized, generation, generative, language model, language models, LLM, LLMs, natural language processing, NLP, random, randomness, tailor, tailored, temperature, text, texts, textual

## Abstract

More clinicians and researchers are exploring uses for large language model chatbots, such as ChatGPT, for research, dissemination, and educational purposes. Therefore, it becomes increasingly relevant to consider the full potential of this tool, including the special features that are currently available through the application programming interface. One of these features is a variable called temperature, which changes the degree to which randomness is involved in the model’s generated output. This is of particular interest to clinicians and researchers. By lowering this variable, one can generate more consistent outputs; by increasing it, one can receive more creative responses. For clinicians and researchers who are exploring these tools for a variety of tasks, the ability to tailor outputs to be less creative may be beneficial for work that demands consistency. Additionally, access to more creative text generation may enable scientific authors to describe their research in more general language and potentially connect with a broader public through social media. In this viewpoint, we present the temperature feature, discuss potential uses, and provide some examples.

## Introduction

ChatGPT [1] is a large language model developed by OpenAI that currently has over 100 million users [2]. As its popularity continues to grow, clinicians and researchers are among many considering its potential applications in health care and academia. In a short time, ChatGPT has been extensively published [3], with clinical researchers exploring its potential utility for a variety of tasks, including answering patient questions [4,5], generating clinical summaries [6], and abstracting data from important documentation (eg, computed tomography reports) [7].

When using ChatGPT, one can interact through the website by providing a single prompt or engaging in a conversation. In addition to this more well-known web-based version of ChatGPT, there is also an application programming interface (API) that allows for more customization and flexibility. With the API, users can programmatically interact with ChatGPT and modify features for their specific use case. Although this approach may currently require more technical expertise for clinicians to use, its features may become available on the web interface in future iterations of the tool. Therefore, these features are important to understand and relevant to discuss in terms of their meaning for clinicians and researchers in advance of their more widespread use. Additionally, they have direct implications for introducing greater reproducibility in use cases where this matters.

## The Temperature Feature of ChatGPT

ChatGPT generates text through a probabilistic language modeling approach, where it writes responses word by word, calculating the most likely next word in the sequence. A key feature that influences this behavior is called temperature [8, 9]. In this context, temperature is a value from 0 to 2 that adjusts how random each subsequent word in the chat output is. A value of 0 will give the most probable word and, thus, the least variability. As the value increases toward and beyond 1, the next word becomes less probable, leading to more randomness and “creativity” in the response. This feature can currently be adjusted in the API, where the default value is 1 [9].

The ability to adjust the “creativity” of ChatGPT output should also be of interest to clinicians and medical researchers using the tool. By accounting for temperature, large language models such as ChatGPT can be tailored for different use cases. Lowering ChatGPT’s creativity level would be preferable for tasks that require more consistent outputs; for clinicians and researchers, tasks of this sort may include summarizing patient data (eg, symptoms and medications) or streamlining administrative tasks (eg, billing inquiries and patient registrations). Alternatively, increasing the creativity level may provide better outputs for less structured tasks and may specifically hold relevance for improving clinicians’ and researchers’ digital communication with other health care workers, patients, and a wider audience.

Currently, many clinicians may find it difficult to engage with the broader public when it comes to disseminating findings. A lack of public accessibility can limit the impact of research. Creativity proves to be a powerful ally in communication, as it simplifies complex concepts, fosters critical thinking, and enhances the accessibility of intricate information. These factors collectively contribute to increased audience engagement and more effective message transmission. This feature has value for developing patient education materials, medical courses, science communication, and public research dissemination [10]. Increasing the creativity level could be useful when trying to report research results on social media [11], at conferences, or in scientific publications. Among other things, researchers could use a more creative ChatGPT to help come up with tags that apply to their work, brainstorm analogies or metaphors for scientific concepts, and check the tone of their writing.

## Illustrating This Feature: Example

As an illustration of the “creativity” feature in ChatGPT, we have provided ChatGPT with the abstract of a recently published article on the development of a keyword library for capturing symptom talk in oncology [12]. While setting the temperature to low (0.0), medium (0.5), and high (1.0), we asked ChatGPT to create a tweet for a public audience, a title for a scientific journal article, and a title for a keynote address. Example ChatGPT-generated responses can be found in Table 1.

**Table 1:** ChatGPT-generated responses on the prompts for the different temperatures. For each temperature level, the same prompts were run 10 times in a ChatGPT model (text-davinci-003).

Temperature	Low (0.0)	Medium (0.5)	High (1.0)
Tweet for public audience	We developed a keyword library to detect symptom talk in transcribed clinical conversations. Our findings suggest that physical symptoms are more easily detected than psychological symptoms, and that more advanced computational models are needed to detect symptom expressions. #AI #Healthcare #SymptomDetection	Have you heard about the new study that developed and evaluated a natural language processing keyword library to detect symptom talk in oncology conversations? The findings highlight the need for more advanced computational models to detect symptom expressions in transcribed clinical conversations. #Oncology #SymptomTalk #NLP #ComputationalMethods	Check out how computational methods can help us detect patient symptoms in clinical conversations! A recent study developed & evaluated a keyword library for capturing expressed symptoms, highlighting the need for more advanced models for real-time detection of symptoms at scale. #AI @healthinformatics
Title for scientific journal	Detecting Symptom Talk in Transcribed Outpatient Oncology Conversations: Developing and Evaluating a Natural Language Processing Keyword Library	Keyword-Based Detection of Symptom Talk in Oncology Conversations: Developing a Natural Language Processing Library and Exploring Opportunities for Advanced Computational Models	Unlocking the Ambiguity of Symptom Talk: Developing and Evaluating a Natural Language Processing Library for Detecting Outpatient Oncology Conversations
Title of a keynote address	Unlocking the Potential of Computational Methods for Detecting Symptoms: Achieving Better Patient Care Through Natural Language Processing	Harnessing the Power of Computational Methods to Detect Patient Suffering: A Study of Symptom Talk in Oncology Conversations	The Ambiguity of Symptom Expressions: Utilizing Computational Methods to Better Attend to Patient Suffering

The examples shown in Table 1 illustrate that when the creativity level in ChatGPT is adjusted, slightly different responses are generated; these different creativity levels may provide more useful output depending on the task at hand. For example, a tweet created with a high level of creativity includes an exclamation mark and directly addresses the audience. Therefore, it may be more engaging compared to tweets with a low or medium creativity level. The title of the article and keynote generated with high “creativity” were more surprising and potentially less useful for these tasks, although this may depend on the context, setting, and personality of the user. For these tasks, the low- and medium-creativity titles were more straightforward. Importantly, these lower values do translate to more consistent responses. We ran each of these prompts 10 times, and at a temperature level of 0, all responses were identical. Given ChatGPT’s normally variable output, this feature holds exciting implications for scenarios where consistency and reproducibility are preferred.

In addition to the results reported above, we have also experimented with adjustments in temperature level using other ChatGPT models (ie, gpt-3.5-turbo-1106, gpt-3.5-turbo-instruct, and gpt-4-1106-preview). All outputs appear in Multimedia Appendix 1**.** In contrast to what we found when using the ChatGPT model “text-davinci-003,” some other models showed some variability, even at a temperature level of 0. Regardless, the relative variability of outputs is still modified by temperature, with a higher temperature increasing creativity. Users should consider and test how temperature impacts outputs within the model they are using.

In the examples provided above, we have demonstrated how adjusting the level of creativity can enhance science communication, making it more engaging. However, it is crucial to also acknowledge the potential risks associated with increasing creativity, especially for clinical cases. Using ChatGPT with high creativity settings in clinical contexts, such as for summarizing patient medical data, can be problematic. Excessive creativity might lead to the embellishment or misrepresentation of crucial information, either by omitting vital details or interpreting data too liberally. Such inaccuracies could impact patient treatment and outcomes. Therefore, it is advisable to lower the creativity level of ChatGPT in clinical applications. By doing so, we ensure that the summarized information remains faithful to the original data, thereby prioritizing accuracy and reliability over creative expression.

In summary, the temperature feature of ChatGPT allows users to adjust the level of “creativity.” Although no previous articles have discussed or investigated this feature for its use in clinical research, it shows promising potential for clinicians and researchers. Both high and low creativity levels could have interesting applications for health care and may broaden the ways clinicians and researchers consider using artificial intelligence (AI) tools to close gaps in areas such as digital communication. ChatGPT documentation suggests using a temperature value of 0 to 0.2 for more focused (less creative) tasks and 0.8 to 1 for more random (more creative) tasks [9]. As large language models are variable and use case dependent, we strongly suggest testing and validating the proper temperature level for your specific use case. While this feature is a powerful tool that could be useful for creating easy-to-understand summaries, captivating social media posts, or making complex information more accessible to a wider audience, the parameters need to be carefully tweaked to find a balance between coherence and creativity and to tailor to specific needs. Looking ahead, as AI continues to advance in the health care sector, the temperature feature can play a pivotal role in health care applications in generative AI, unlocking the potential for more accurate, empathetic, or creative interactions between AI and health care stakeholders.

## Appendix

Multimedia Appendix 1 All data presented in this article, ChatGPT outputs for tests of 3 prompts across 3 temperature values for 3 different models (gpt-3.5-turbo-1106, gpt-3.5-turbo-instruct, and gpt-4-1106-preview; 100 runs for each test), and a summary document describing the multiple model tests.

## Abbreviations

AI: artificial intelligence
API: application programming interface

## Data Availability

All data generated or analyzed during this study are included in this published article and Multimedia Appendix 1.
