# Supplementary material for: The Temperature Feature of ChatGPT: Modifying Creativity for Clinical Research
Source: JMIR Hum Factors. 2024 Mar 8;11:e53559. doi: 10.2196/53559 (PMC10960206; doi:10.2196/53559)
Supplement: Multimedia Appendix 1 [file humanfactors_v11i1e53559_app1.zip › Appendix/Temp_Variability_Analysis.docx]

**Supplemental Data**

Detail: Additional Analysis

*Overview*

We performed nine tests to observe variability among ChatGPT outputs for the same prompts using different models at different temperatures.

Prompts:

1. “Create a tweet for a public audience of the abstract [Abstract]”
2. “Create a title for a scientific journal article of this abstract [Abstract]”
3. “Create a title of a keynote address for this abstract [Abstract]”

Models:

1. gpt-3.5-turbo-instruct
2. gpt-3.5-turbo-1106
3. gpt-4-1106-preview

Temperature Values:

1. 0 (low creativity)
2. 0.5 (“medium” creativity)
3. 1 (high creativity)

*Analysis*

For each test, we ran the prompt 100 times through the API and saved all outputs to a csv file. Output dissimilarity was calculated using Jaccard Distance:

$$d_{J}\left( A,B \right)=\frac{\left| A\cup B \right|-|A\cap B|}{|A\cup B|}$$

This calculation can be used to compare the sets of words used in different data samples. To get these sets, outputs were tokenized using the NLTK TweetTokenizer function (in the case of outputs of prompt 1) or by splitting on whitespace (in the case of outputs of prompts 2 and 3).

*Results*

The following are Jaccard Distance values calculated for each test in aggregate:

| **Model_prompt** | **Temperature** | | |
| --- | --- | --- | --- |
|  | **0** | **0.5** | **1** |
| gpt-3.5-turbo-instruct_tweet | 0 | 0.642288 | 0.709613 |
| gpt-3.5-turbo-instruct_title | 0 | 0.363676 | 0.577506 |
| gpt-3.5-turbo-instruct_keynote | 0 | 0.623068 | 0.766099 |
| gpt-3.5-turbo-1106_tweet | 0.500523 | 0.602072 | 0.661758 |
| gpt-3.5-turbo-1106_title | 0.090821 | 0.211031 | 0.407294 |
| gpt-3.5-turbo-1106_keynote | 0.184375 | 0.488759 | 0.703661 |
| gpt-4-1106-preview_tweet | 0.32965 | 0.612816 | 0.710919 |
| gpt-4-1106-preview_title | 0.020202 | 0.167054 | 0.416569 |
| gpt-4-1106-preview_keynote | 0.402625 | 0.639283 | 0.737705 |

We visualized these values for each model using simple bar plots.


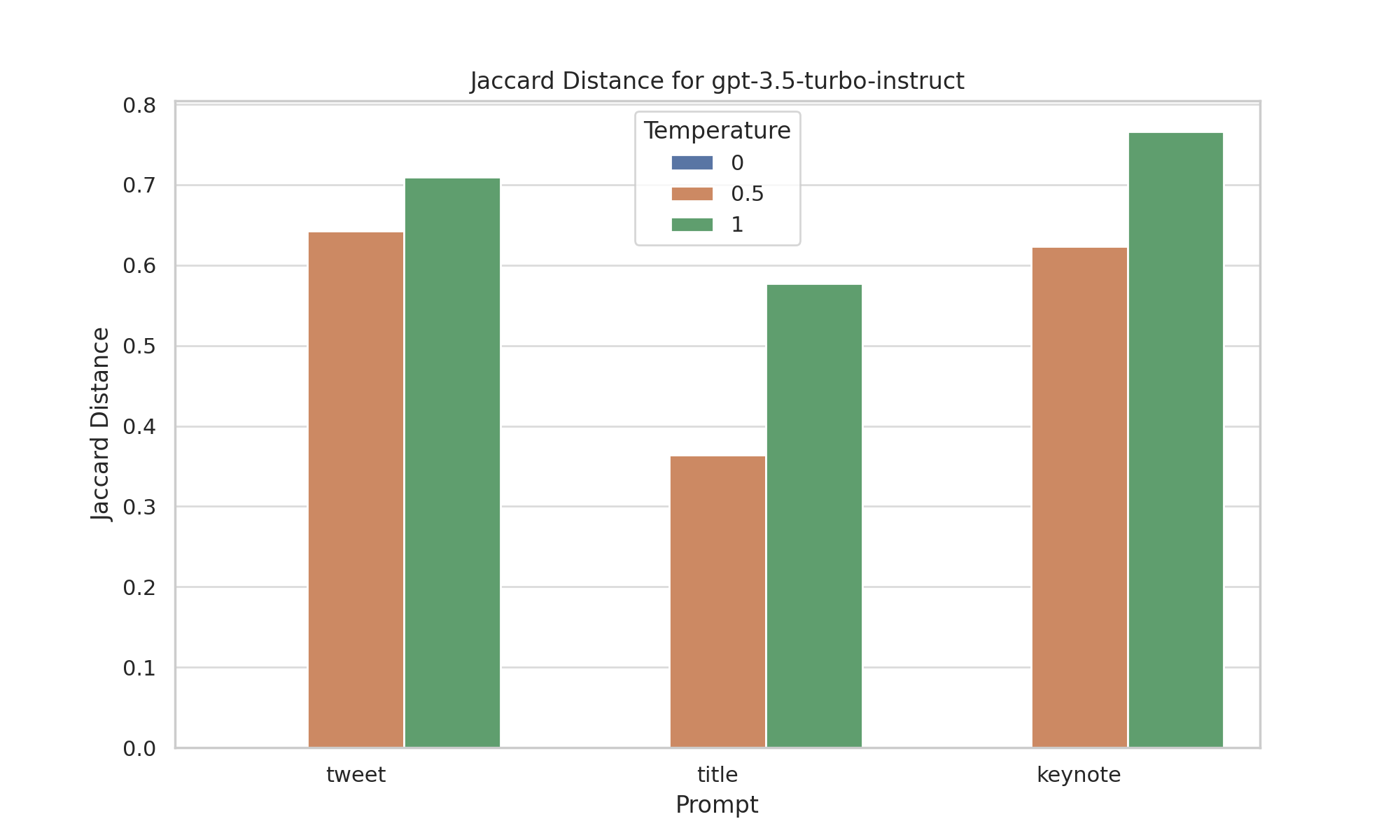


In the case of the gpt-3.5-turbo-instruct model, we observed no dissimilarity between word sets at a temperature of 0. As temperature increased, so did Jaccard Distance.


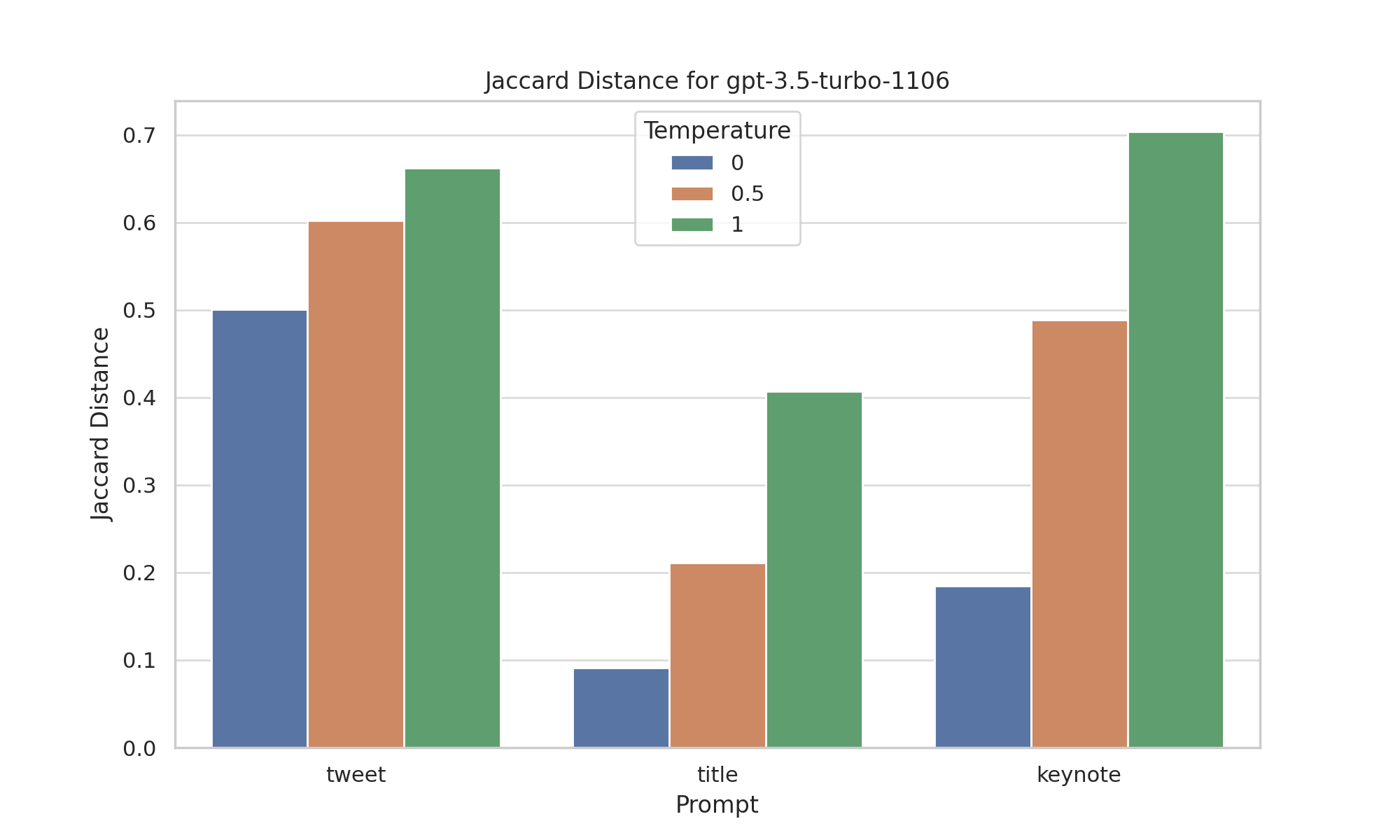


With the gpt-3.5-turbo model, we observed dissimilarity at all temperature levels. As observed with the gpt-3.5-turbo-instruct model, dissimilarity did still increase as temperature was increased.


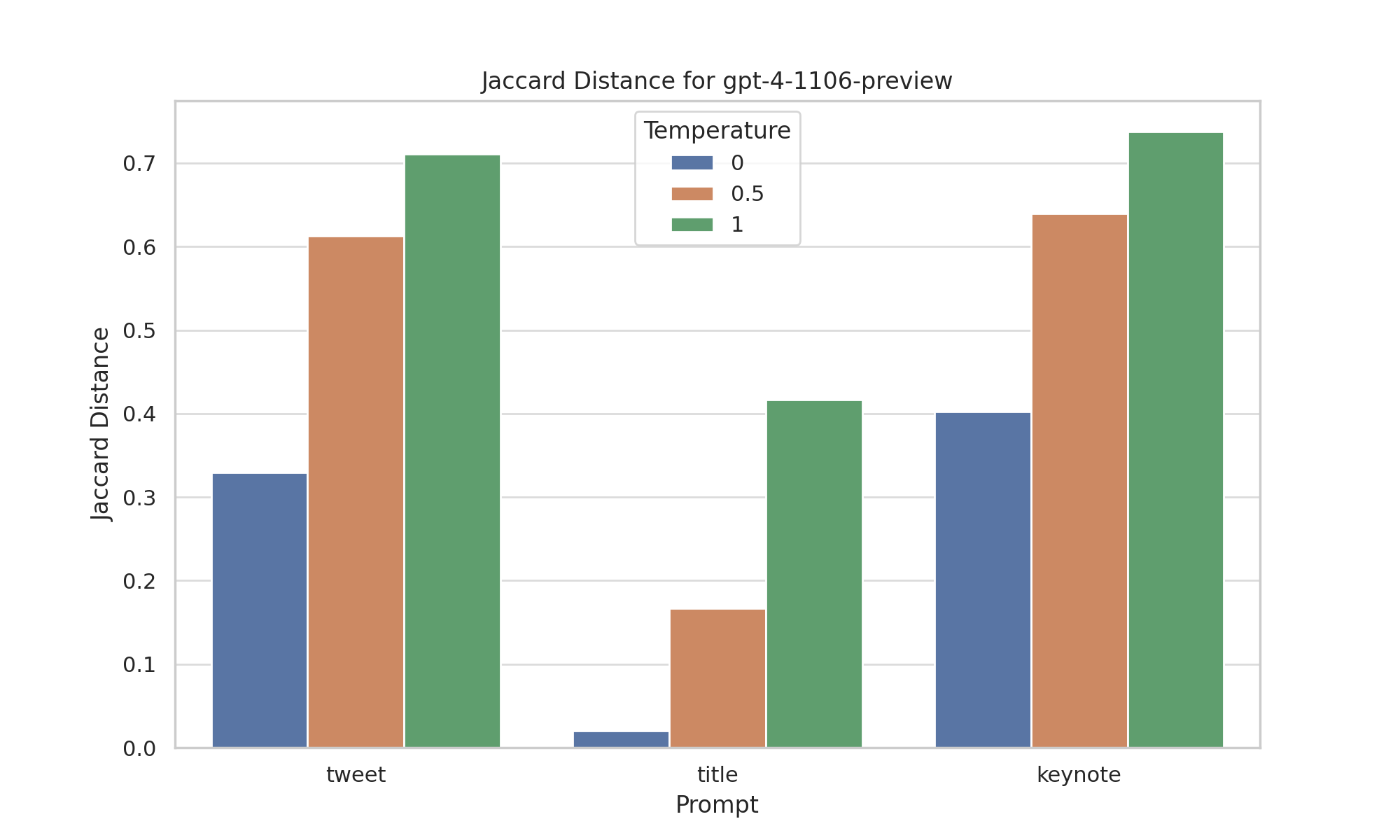


Using the gpt-4 model, a similar pattern was seen; depending on the prompt being tested, there was dissimilarity of differing levels at a temperature of 0. As with the other models, dissimilarity increased with temperature increasing.

*Observations/Discussion Points*

Though level of dissimilarity at a temperature of 0 varies across models, a pattern of dissimilarity increasing with temperature can be observed. The level of variability/dissimilarity seen in outputs at a temperature of 0 differs depending on the prompt.
